# Cranial Subdural Empyema Due to *Citrobacter koseri* in a 96‐Year‐Old Immunocompromised Patient: Case Report

**DOI:** 10.1155/cris/8718830

**Published:** 2026-09-30

**Authors:** Milica Malesevic-Lepir, Mohamed M. A. Mohamed, Sali Binali, Alexander Piotrowski, Benjamin Voellger

**Affiliations:** ^1^ Department of Neurosurgery Hospital Bad Hersfeld Bad Hersfeld Germany; ^2^ Department of Neurosurgery Sana Hospital Offenbach Offenbach Germany; ^3^ Department of Neurosurgery Zagazig University Zagazig Egypt zu.edu.eg; ^4^ Department of General Surgery Hospital Bad Hersfeld Bad Hersfeld Germany; ^5^ Department of Anesthesiology and Intensive Care Hospital Bad Hersfeld Bad Hersfeld Germany; ^6^ Department of Urology Hospital Bad Hersfeld Bad Hersfeld Germany; ^7^ Department of Neurosurgery Marburg University Marburg Germany; ^8^ Department of Neurosurgery St. Vincenz Hospital Paderborn Paderborn Germany

**Keywords:** case report, *Citrobacter* spp., corticosteroids, pemphigus, subdural empyema

## Abstract

Subdural empyema (SDE) is a life‐threatening condition. Reports on SDE are scarce. We report the case of a 96‐year‐old immunocompromised patient on steroids for pemphigus who received repeat burrhole trephination and antibiotics to treat SDE. We identified *Citrobacter koseri* as the causative pathogen. Our patient made a good recovery at 2 months. We conclude that surgical treatment may yield a favorable outcome after cranial SDE, even in an elderly immunocompromised patient.

## 1. Introduction

Subdural Empyema (SDE) is a life‐threatening [1] condition characterized by a collection of pus between the dura and arachnoid mater. SDE may lead to rapid neurological deterioration, coma, and death. Treatment options comprise antibiotic therapy, surgical evacuation [1], and optimum therapy of comorbidities.

Cranial SDE typically occurs secondary to sinusitis, otitis, or head trauma [2]. Hematogenous spread from a distant site of infection [3] appears to happen less often. Immunocompromised patients may be particularly prone to develop SDE. However, a dysfunction of the immune system may impair wound healing and increase the risks inherent in therapy, such as allergies or other adverse drug reactions (ADRs).

Here, we report on the case of a 96‐year‐old immunocompromised patient with cranial SDE whom our interdisciplinary team treated surgically with success.

## 2. Case Presentation

A 96‐year‐old male with pemphigus vulgaris on chronic steroid therapy (prednisolone 5 mg daily) presented from a nursing home to the emergency room. Recently, he had developed right‐sided hemiparesis (Janda grade 2–3) and severe global aphasia. Initial cranial computerized tomography (CCT, Figure 1a) revealed a space‐occupying subdural fluid collection over the left hemisphere with a midline shift of 6 mm to the right.

**Figure 1:**
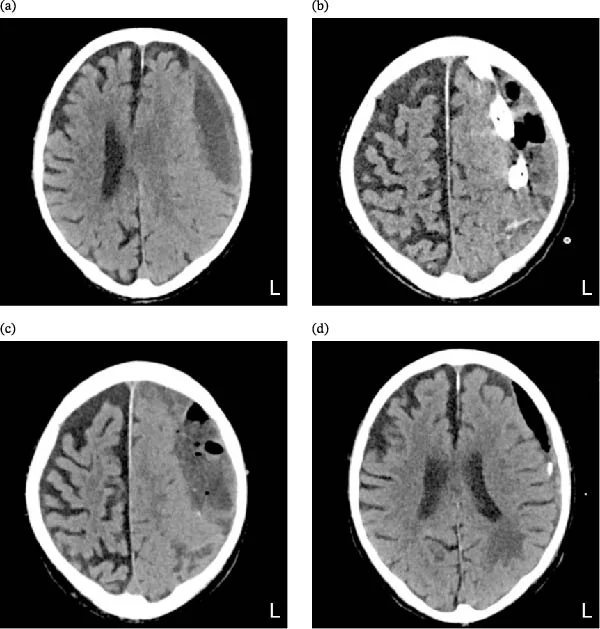
Cranial computerized tomography (a) prior to 1st surgery, a space‐occupying subdural collection on the left with compression of the left hemisphere and midline shift to the right; (b) after 1st surgery, a remnant of the subdural collection on the left, with some air pockets and a subdural drainage alongside the frontal lobe as well as a thin blood layer on the parietal lobe; (c) prior to 2nd surgery, the remnant of the subdural collection still considerable in size, with small air pockets and a significant compression of the left hemisphere; (d) after 2nd surgery, a marked decrease in brain compression, midline shift and residual subdural collection, along with a thin air pocket and a subdural drainage on the left. L, left.

The white blood cell (WBC) count (17.2 × 10^9^/L (reference range: (4.4–11.3) × 10^9^/L), for the timeline of laboratory findings see Figure 2) and c‐reactive protein (CRP) plasma level (165 mg/L (reference range: less than 5 mg/L)) were elevated. Considering the patient’s history and laboratory results, we suspected that the subdural collection was an SDE. The patient underwent urgent left parietal burr hole trephination, subdural irrigation, and placement of a subdural drainage under general anesthesia on the day of admission. Intraoperatively, we evacuated pus from the subdural space (for the timeline of the clinical course, see Figure 2).

**Figure 2:**
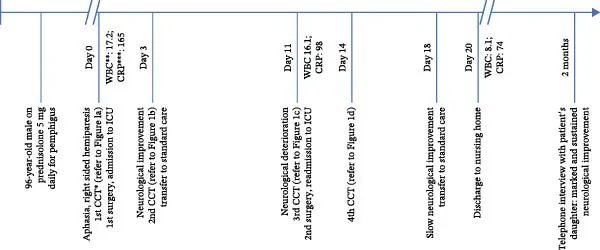
Timeline of the patient’s history, clinical findings, imaging results, interventions, and laboratory results.  ^∗^CCT, cranial computerized tomography,  ^∗∗^WBC, white blood cells (reference range: (4.4–11.3) × 10^9^/L), and  ^∗∗∗^CRP, C‐reactive protein (reference range: <5 mg/L).

After surgery, the patient’s aphasia and hemiparesis improved. The patient started meaningful verbal communication. He was partially orientated, and he was able to use his right hand. Follow‐up CCTs on days 0 and 3 (Figure 1b; for the timeline of CCT scans, see Figure 2) after surgery showed a decrease in midline shift, a remnant of the subdural fluid collection, and a thin cortical blood layer. The patient was transferred from the intensive care unit (ICU) to standard care on day 3.

Steroid treatment was halted due to the newly diagnosed life‐threatening infection of the subdural space. Cultivation of intraoperative swabs revealed *Citrobacter koseri*, and the patient received intravenous antibiotics (meropenem and cefotaxim) according to the resistogram. We regularly irrigated the subdural space with sterile isotonic sodium chloride solution until we removed the drainage. Urinary retention prompted urological evaluation, which confirmed pyuria from *C. koseri*. We treated this with a bladder irrigation catheter.

On day 11, worsening of aphasia and hemiparesis led to a repeat CCT scan (Figure 1c), showing that the remnant of the subdural collection was still considerable in size. The WBC count (16.1 × 10^9^/L) and CRP level (98 mg/L) were still not within normal ranges. We obtained informed consent for repeat surgery from the patient’s daughter, who is also his legal representative.

Subsequently, extension of the left parietal burr hole in a cloverleaf fashion, irrigation, and drainage placement were performed under general anesthesia. During this procedure, large amounts of pus were evacuated again, followed by the resection of the empyema’s capsule. Follow‐up CCT on day 14 (Figure 1d) showed marked decreases in the midline shift and size of the subdural collection. Again, we irrigated the subdural space regularly with isotonic sodium chloride solution until we removed the drainage. Further laboratory findings included hypokalemia, treated with substitution of potassium; hyperglycemia, treated with insulin; and yeast within the urinary tract, treated with fluconazole.

We interpreted intermittent lacks of responsiveness as absence seizures and treated them with levetiracetam. At that point, the patient’s daughter declared any further escalation of treatment to be not compatible with the patient’s presumed will. The patient was again transferred from the ICU to standard care on day 18.

After the second surgery, the WBC count normalized (8.1 × 10^9^/L, day 20), and the CRP level was maintained (74 mg/L, day 20). The patient slowly recovered from aphasia and hemiparesis. On day 20, the patient was discharged to his nursing home. At discharge, he responded to simple questions adequately with “yes” or “no,” while the right‐sided hemiparesis had slightly improved. At discharge, the antibiotic regimen was switched to oral ciprofloxacin for another 6 weeks.

Follow‐up telephone interviews 2 weeks after discharge with the staff at the nursing home and 3 and 6 weeks after discharge with the patient’s daughter confirmed a sustained neurological improvement: nursing staff and family reported that the patient had almost fully recovered from hemiparesis, communicated verbally in a meaningful way, and that he was able to take in food. The scar over the left convexity was always reported to be in good condition.

The patient had developed diarrhea 5 weeks after discharge. We considered this an ADR to antibiotic treatment and accordingly recommended halting antibiotics at the last telephone interview.

## 3. Discussion

SDE is a neurosurgical emergency. It bears significant mortality if untreated [1]. While neurosurgeons have considered urgent surgical evacuation of pus, either via burr hole drainage or open surgery, and antibiotics as cornerstones of SDE treatment [1] for decades, scientific evidence is scarce and actually warrants a systematic literature review. This, however, would exceed the scope of the case report as published here.

Sometimes, infection control requires multiple procedures, as seen in our case and reported in the literature [4]. Following surgery, early microbiological diagnosis guides antibiotic therapy, which in turn determines the outcome [1].

*Citrobacter* spp. are facultative anaerobic Gram‐negative rod‐shaped bacteria involved mainly in neonatal meningitis [5]. However, they may also account for central nervous system (CNS) infections in adults, especially in cases with immunosuppression [6, 7]. As in our case, they may arise from the urinary tract [6].

Sometimes, it is difficult to differentiate infectious disease from neoplasm [8] or hemorrhage prior to or during cranial surgery. The rarity of cranial SDE underscores the need for high suspicion, particularly in immunocompromised patients presenting with neurological deficiencies and signs of infection.

Oral corticosteroids to treat pemphigus vulgaris may have considerable side effects [9]. We therefore decided to halt prednisolone in our patient when we diagnosed him with SDE. This decision may, however, increase the patients’ risk of suffering from pemphigus in the future. Multiple comorbidities may further complicate management, as dementia and urinary tract infections did in our patient.

There are only a small number of reports on spinal SDE available. Spinal SDE, like cranial SDE, involves the CNS, albeit with a different set of clinical presentations and surgical approaches.

We conclude that targeted antibiotics, tailored management of comorbidities, and surgical treatment may yield a favorable outcome after cranial SDE, even in an elderly immunocompromised patient.

**Nomenclature**
ADR: Adverse drug reaction
CCT: Cranial computerized tomography
CNS: Central nervous system
CRP: C‐reactive protein
ICU: Intensive care unit
SDE: Subdural empyema
spp.: Species
WBC: White blood cell.

## Author Contributions

Conceptualization, methodology, software, validation, formal analysis, resources, data curation, visualization, supervision, project administration: Benjamin Voellger. Investigation, writing – original draft preparation: Milica Malesevic‐Lepir, Benjamin Voellger, and Mohamed M. A. Mohamed. Writing – review and editing: Sali Binali and Alexander Piotrowski.

## Funding

Open Access funding provided by the Open Access Publishing Fund of Philipps‐Universitaet Marburg.

## Disclosure

All authors have read and agreed to the published version of the manuscript.

## Ethics Statement

Ethical review and approval were waived for this study due to its retrospective, anonymized design.

## Consent

Written informed consent prior to the anonymized publication of the case report has been obtained from the patient’s legal representative.

## Conflicts of Interest

The authors declare no conflicts of interest.

## Data Availability

The data that support the findings of this study are available from the corresponding author upon reasonable request.
